# The Action Representation Elicited by Different Types of Drug-Related Cues in Heroin-Abstinent Individuals

**DOI:** 10.3389/fnbeh.2018.00123

**Published:** 2018-07-02

**Authors:** Hong Zeng, Dequan Su, Pengfei Wang, Mengcheng Wang, Sabine Vollstädt-Klein, Qi Chen, Haosheng Ye

**Affiliations:** ^1^The Research Center of Psychology & Brain Science, Department of Psychology, Guangzhou University, Guangzhou, China; ^2^Department of Addictive Behaviour and Addiction Medicine, Central Institute of Mental Health, Heidelberg University, Mannheim, Germany; ^3^School of Psychology, South-China Normal University, Guangzhou, China

**Keywords:** drug addiction, drug-related cues, DLS-SM system, habitual drug use, fMRI

## Abstract

Drug related cue-induced reactivity plays a significant role in maintaining drug use and relapse in addicted individuals. The activation of Dorsolateral striatum-Sensorimotor system (DLS-SM) has been suggested as an important route through which drug cues may induce automatic drug using behavior. The current study used fMRI to investigate the reactivity of heroin abstinent individuals to different types of cues, to clarify the characteristics of the cues that induce the activation of the sensorimotor area. Forty heroin-dependent abstinent individuals and 29 healthy subjects were recruited to perform the heroin cue-reactivity task during fMRI. The participants’ subjective craving and physical signs were evaluated before and after scanning. Whole-brain analysis showed that compared to drug use tool and drug cues, cues related to drug use action were more likely to activate posterior central gyrus, para-hippocampus, supra marginal gyrus, superior parietal lobule (SPL) and inferior parietal lobule (IPL). These areas are involved in motor preparation and output, indicating that the sensorimotor area is also an important neural basis of craving and automatic drug using behavior, and may mediate craving and drug seeking behavior. Our findings thus suggest that cues related to drug using action may induce automatic drug seeking behavior more than cues related only to the drug itself.

## Introduction

Drug addiction is characterized by compulsive drug taking behavior and high rates of relapse even after many years of abstinence (O’Brien et al., 1977). Exposure to drug-associated cues instigates physiological, behavioral and subjective reactions. This phenomenon, called cue-induced reactivity, includes craving and automatic drug using behavior, in which behavior becomes autonomous and can be performed with little attention, intention, or cognitive effort, constituting a “habit” (Knowlton, 2014). Autonomous behavior is thought to play a significant role in triggering addiction and relapse in drug users.

The majority of the neuroscience research on drug cue-induced reactivity and its neuronal underpinnings has focused on the mesocorticolimbic system (Grant et al., 1996; Brody et al., 2002; Due et al., 2002; David et al., 2005), including the ventral striatum (VS), extended amygdala, hippocampus, anterior cingulate cortex (ACC), prefrontal cortex (PFC) and insula, which are innervated by dopaminergic projections predominantly from the ventral tegmental area (VTA; Nestler, 2005). Whilst OFC is believed to play a key role in reward values, and about the current state and needs of the organism, in order to guide motivated behavior (Goldstein and Volkow, 2011; Luijten et al., 2011; Lucantonio et al., 2012). The ACC is engaged in a range of cognitive tasks, particularly tasks that involve executive function (cognitive control, conflict, or error monitoring; Goldstein and Volkow, 2002; Dosenbach et al., 2006; Cabral et al., 2014). However, it has long been suggested that there is another route through which drug cues may induce increased drug use, which is the activation of conditioned sensorimotor associations (Tiffany, 1990). Drug cues may induce drug using behavior by activating the corresponding brain regions related to action in heroin abstinent individuals (Yalachkov et al., 2010). There is also considerable evidence that the dorsolateral striatum-sensorimotor circle (DLS-SM) is gradually engaged to underlie well established habitual drug seeking behavior (Belin et al., 2009; Vollstädt-Klein et al., 2010; Corbit et al., 2012; Barker and Taylor, 2014; Everitt, 2014).

Recent studies on substance users have offered an interesting perspective on this issue. These studies found that for smokers, areas that were activated in response to the cues included not only the dorsal ACC, OFC and DLPFC, brain areas known for their role in the components of addiction, which are frequently observed in drug cue reactivity researches, but also the anterior intraparietal sulcus (aIPS), left inferior frontal gyrus (IFG) and premotor cortex (PMC), which store and process action presentations (Yalachkov et al., 2009; Stippekohl et al., 2010; Wagner et al., 2011). aIPS and IFG regions were more strongly activated in smokers when they observed other smokers than nonsmokers (Wagner et al., 2011). In other words, smoking cues activated corresponding action representations of addiction in smokers’ brains (Yalachkov and Naumer, 2011), which may indicate that the sensorimotor cortex is involved in the cue-induced reactivity.

Psychologists have theorized that observing action increases the likelihood that a person performs those actions (Boy et al., 2010). Some early neuroscience research also showed that observing related actions can recruit the superior parietal and lateral PFC and influence the link between cues and behavioral responses (Decety et al., 1994; Buccino et al., 2004; Hamilton and Grafton, 2006). Furthermore, action observation also activates similar brain regions as observed in motor imagery, such as the inferior parietal lobule (IPL) and the PMC (Zacks, 2008; Caruana et al., 2014). In addition, manipulable object cues were observed to activate the inferior, middle frontal gyrus and superior parietal lobule (SPL; Lewis, 2006; Nachev et al., 2008; Sumner and Husain, 2008). All of the research results may reflect the engagement of automatized motor schemata and action knowledge in drug use behavior under drug related cues.

However, these findings are limited to smokers and alcoholics, and it remains untested whether and to what extent the action representation works on other types of substance use individuals. In addition, researchers do not know if there are any differences in reactivity to different types of drug-related cues or the exact role and function of different drug-related cues, such as images of drugs, drug use action and drug use tools, in drug-related cue reactivity. Clarification of these issues would further deepen the understanding of drug addiction in terms of cue-induced reactivity and its underlying mechanisms. Thus, the detailed and precise study of the relevance of brain regions for drug cue reactivity in response to different cues, particularly the study of the link between the activation of action-related brain regions and different types of drug-related cues, is essential.

Based on our previous researches (Zeng et al., 2015; Su et al., 2016), the current study used fMRI to investigate the brain reactivity of heroin dependent individuals under different types of cues, to identify the basic mechanism of cue-induced reactivity and what aspect of the cues would most activate the action representation and influence the cue-induced reaction. We hypothesized that all cues related to heroin would activate the brain areas associated with reward, motivation, and executive function. Moreover, the cues related to drug use action or tools would activate more of the sensorimotor regions and DLS. Both reward and DLS-Sensorimotor brain systems would be activated in the addicted brain in response to the drug-related cues.

## Materials and Methods

### Subjects

Forty (26 males and 14 females) heroin abstinent individuals and 29 controls (19 males and 10 females) with no history of any drug addiction were recruited to participate in the study at a volunteer drug rehabilitation center and factories in Guangdong Province, China. All the participants were required to be from 18 years to 45 years old and to have at least 6 years education (graduated from primary school). No history of major psychiatric disorders (e.g., schizophrenia or mania) according to DSM-IV or serious head injury or neurological disorder was reported. None was taking medications known to affect the central nervous system (e.g., tranquilizers or hypnagogs).

All the heroin abstinent individuals were required to conform to the dependance standard of DSM–IV and to have been abstinent for at least 1 month, with no alcohol consumption at least 1 day before the experiment (cigarettes were allowed). To make sure the subjects were in the detoxification phase, all of them had urine testing and all tests were negative for heroin.

A questionnaire designed for this study was used to assess age, gender, years of education and drug use behavior. The Self-report Anxiety Scale (SAS; Zung, 1971) was used to evaluate the severity of anxiety. Drug craving before and after scanning was assessed by using a visual analog scale (VAS) on which the participants had to rate their current craving for heroin. Heart rate, respiration, blood pressure, galvanic skin response (GSR) and skin temperature were also taken before and after scanning as additional measures of cue reactivity. Wrist blood pressure monitor was used to measure heart rate and blood pressure. Measure length is 1 min. Aural thermometers was used to measure the temperature, the length is 2 s. Galvanic skin sensor was used to measure the GSR. We report the data of 30th seconds.

The protocol was approved by the South China Normal University Committee. All participants gave informed consent in accordance with the Declaration of Helsinki and with the guidelines set for MRI scan by the committee for the protection of human subjects. Each subject received 200 Chinese Yuan for participating in the experiment.

### Stimuli

**Figure 1:**
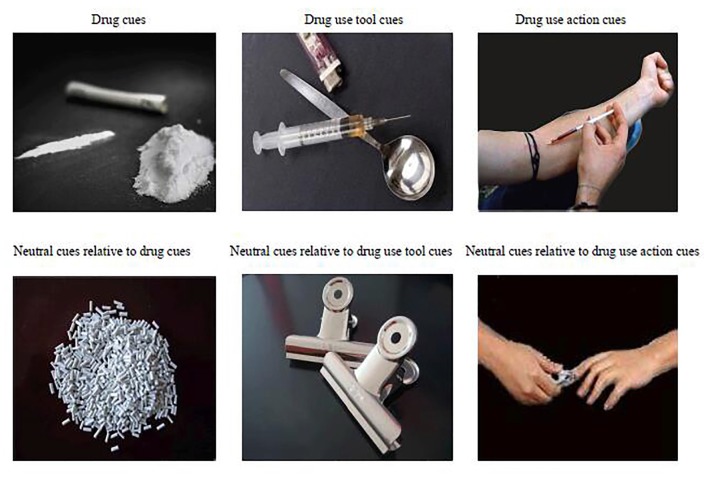
Drug related pictures and relatively neutral ones.

Prior to the study, a pilot study was conducted to identify pictures that would evoke significant craving in heroin addicts. Two-hundred and one drug-related pictures were collected and divided into three types: (1) drug images containing only the drug itself, not displaying tools or any actions; (2) drug use tool images consisting of pictures of a syringe and other tools for using heroin; and (3) drug using action images containing people engaged in the activities of using tools to absorb or inject heroin. The pictures did not show full figures of people, but rather showed arms and hands engaged in the action; see Figure 1.

The neutral stimuli pictures were taken by ourselves and were also divided into three types, each of them corresponding to drug-related stimuli respectively: granular material pictures; daily life manipulable object pictures and pictures of people engaged in activities using manipulable objects to do something on their body. We also tried to match the action between the neutral and drug use pictures. In addition, all drug-related stimuli pictures and neutral pictures were matched in size, quality and layout.

After all the pictures were processed, 30 heroin abstinent individuals (who were different participants from those in the fMRI study: mean age = 39.27 ± 5.37 years, education years = 10 ± 2.42 years, length of heroin dependance: 11 ± 5.68 years, length of abstinence: 19.3 ± 15 months) from a volunteer detoxification center were invited to evaluate the degree of craving elicited by the different cue pictures. A VAS (0–7) was used to score the craving. One–way Analysis of Variance indicated the main effect of cue type as the predictor of craving was significant, *F*_(2,28)_ = 6.96, *p* < 0.001. Drug-related pictures that were scored less than 3 were deleted. In the end, we had 45 drug cue pictures, 45 drug use tool pictures, 45 drug use action pictures and relatively neutral pictures.

### The Experiment Design and Process

A 2 (subjects group: Heroin Abstinent Group (HAG) vs. Health Control Group (HCG)) × 2 (cue category: drug-related vs. neutral) × 3 (cue type: drug vs. drug use tool vs. drug use action) block design was employed in the fMRI task. There were six experimental conditions for each subject and 45 trials for each condition. To prevent fatigue and to ensure optimal fixation, 45 trials of each condition were split into nine blocks. Each 15 s block consisted of five trials, each of which showed a picture for 2 s. A cross hair was presented for 500 ms, before and after each trial. In total, there were 54 blocks (9 × 6 conditions), which were presented in a pseudo-randomized order (avoiding two successive presentations of the same condition). Before and after each block, there was a 1200 ms interval. The whole experiment was 664 repetition time (TR), 24.3 min.

While lying in the scanner, participants held a response pad in their hand. The stimuli were presented through a LCD projector onto a rear projection screen located behind the participants’ head. Participants viewed the screen through an angled mirror on the head-coil and were asked to press the button to tell that they saw the picture clearly. Stimulus presentations were delivered using the E-Prime software package (Psychology Software Tools, Inc., Pittsburgh, PA, USA).

### MRI Data Acquisition

MRI data acquisition was performed using a 3-Tesla Siemens scanner with a 32-channel head coil (Siemens, Erlangen, Germany). For functional imaging, echo planar imaging (EPI) images were acquired using a T2*-weighted gradient echo sequence with a TR of 2200 ms, an echo time (TE) of 30 ms, a flip angle (FA) of 90°, a field of view (FOV) of 220 × 220 mm and a matrix size of 64 × 64. Each EPI image comprised 36 axial slices of 3-mm thickness, voxel size = 3.1 × 3.1 × 3.0 mm^3^. In addition, a high-resolution structural brain image was acquired using a three-dimensional T1-weighted MP-RAGE sequence with a TR of 2300 ms, a TE of 3.24 ms, an FA of 9°, an FOV of 256 × 256, a matrix size of 256 × 256, and 192 axial slices of 1-mm slice thickness.

### Data Analysis

For each participant, the acquired MRI images were preprocessed using SPM12^1^. First, slice timing correction was performed, and all EPI volumes were spatially realigned for motion correction and coregistered to the participant’s T1-weighted structural image. Second, the structural image was segmented into gray matter, white matter and cerebrospinal fluid and normalized to Montreal Neurological Institute (MNI) space, using the CAT12 toolbox^2^. Third, using the deformation field obtained through the normalization process, the coregistered EPI volumes were normalized to MNI space and resliced to 2-mm isotropic voxels. Finally, the normalized EPI volumes were spatially smoothed with an isotropic 8 mm full-width-at-half-maximum Gaussian kernel.

The preprocessed volumes were entered into a voxel-wise general linear model (GLM) to identify task-related activation for each participant. The GLM included a separate regressor for each stimulus condition: drug pictures (Drug), drug use tool pictures (Tool), drug using action pictures (Action) and neutral pictures (C_Drug), neutral tool pictures (C_Tool), neutral action pictures (C_Action). Each regressor was generated by convolving a canonical hemodynamic response function into a boxcar function representing stimulus presentation. A temporal high-pass filter with a cutoff of 128 s was also incorporated into the GLM for baseline correction. Additionally, the six head movement parameters derived from the realignment procedure were included as covariates of no interest.

At the group level, the six first-level individual contrast images of the HAG and the six first-level individual contrast images of the HCG were then fed into a 2 (subjects group) × 2 (cue category) × 3 (cue type) ANOVA employing a random-effects model. Areas of activation of main effects and interaction effects were identified as significant only if they passed a conservative threshold of *p* < 0.001, corrected for multiple comparisons (family-wise error correction, FWE) at the cluster level with an underlying voxel level of *p* < 0.001, uncorrected. Parameter estimates for the BOLD responses in the peak voxels of the brain regions significantly activated were further extracted using MarsBaR 0.43^3^ and examined by planned *t*-tests (with Bonferroni’s correction).

## Results

### Demographic Data

Forty heroin abstinent dependent individuals were screened to participate in the experiment. Data from three persons were discarded due to movement. We finally obtained 37 participants (24 males and 13 females) in the heroin abstinent dependent group (HAG) and 29 in the HCG. All of the individuals in HAG had been abstinent from heroin at least for 1 month; see Table 1.

**Table 1:** The demography data of the participants (*n* = 66).

	*HAG(37)*		*HCG(29)*		Cohen’s d
	*M*	*SD*	*M*	*SD*	
Age	41.79	2.36	44.01	4.87	−0.58
Years of education	9.83	1.21	10.41	1.16	−0.49
Cigarettes (each day)	14.66	6.54	9.76	8.55	0.64
Alcohol (ml/day)*	21.89	7.89	10.28	9.77	1.31
Anxiety level*	30.32	6.28	27.88	4.79	0.44
Duration of dependance (months)	211.32	47.32			
Dosage (grams/day)	0.46	0.18			
Duration of abstinence (months)	42.11	21.65			

*p < 0.05; HCG, Healthy control group; HAG, Heroin abstinent group.

### Self-Reported Craving Level and Physical Signs Before and After Scanning

Before and after the scan, all the participants were tested for craving level by self–report and for physical signs such as heart rate and temperature. Paired–samples *t* tests were used to compare pre-scan and post-scan measures. The results showed there were significant differences for temperature and heart rate, but no significant differences for self-reported craving level or other physical signs between pre–scan and post–scan. This may be due to the insufficient difference in craving levels between pre-scan and post-scan, which failed to induce a difference in these psychophysiological indexes. Heart rate decrease after scan may be because of the tension release after the scan was completed; see Table 2.

**Table 2:** The difference in physical signs and craving level before and after scan.

	Pre-Scan	Post-Scan	*t*	*P*	Cohen’s d
Craving level	3.39 (1.55)	3.48 (1.50)	0.36	0.71	−0.06
Heart rate (beats/min)	76.33 (10.66)	72.97 (11.68)	2.38	0.03	0.30
Skin temperature	36.57 (0.28)	36.82 (0.27)	−2.03	0.04	−0.91
GSR (30 s)	0.67 (0.12)	−2.41 (1.79)	0.97	0.33	2.42
Systolic pressure SBP (mm/Hg)	121.55 (15.21)	129.01 (12.67)	0.18	0.84	−0.53
Diastolic pressure DBR (mm/Hg)	82.55 (11.24)	82.69 (10.33)	−0.57	0.58	−0.01

*GSR, galvanic skin response*.

### fMRI Results

#### Main Effect of Subjects Group, Cue Category and Cue Types

**Figure 2:**
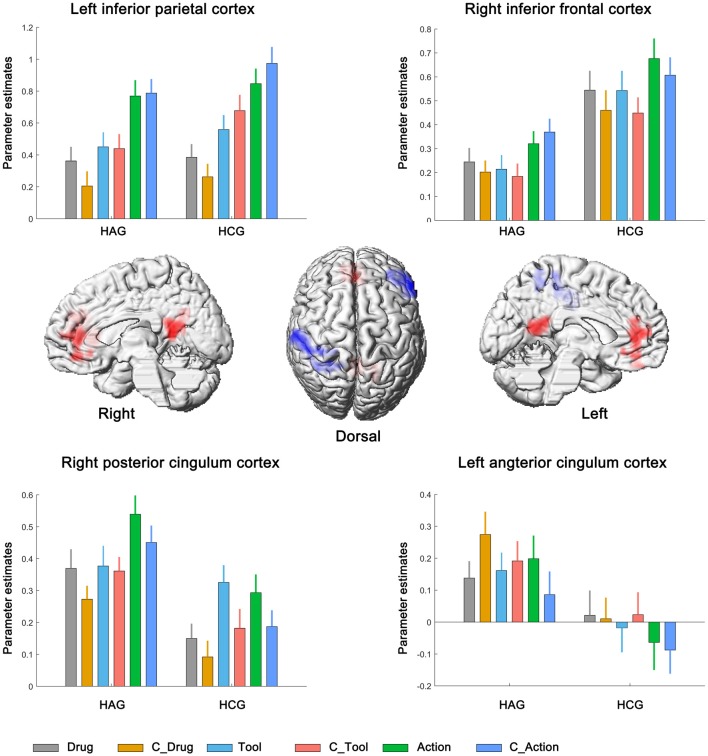
Neural correlates underlying the main effect of subjects group. HAG, Heroin abstinent group; HCG, Health control group; C_Drug, neutral drug pictures; C_Tool, neutral tool pictures; C_Action, neutral action pictures.

We first identified brain regions associated with the main effect of subjects group. Right inferior frontal cortex (IFC) and left inferior parietal cortex (IPC) showed significantly lower neural activity in HAG group than in HCG group. Besides, left anterior cingulum cortex (ACC) and right posterior cingulum cortex (PCC) showed significantly higher neural activity in HAG group than in HCG group; see Figure 2, Table 3A.

**Table 3:** Brain regions showing significant relative increases of BOLD response associated with the effect of subjects group, cue type and cue category.

Brain regions	L/R	Cluster size Voxel	BA	Peak location			*Z*-Score
				X	Y	Z	
**(A) main effect of subjects group**							
Inferior frontal cortex	R	227	45	57	27	12	7.81
Inferior parietal cortex	L	254	2	−51	−36	57	7.59
Posterior cingulum cortex	R	205	23	12	−51	15	6.10
Anterior cingulum cortex	L	219	11	−6	36	6	5.24
**(B) main effect of cue category**							
Lingual	R	1480	37	21	−48	−9	5.24
*Lingual*	*L*		*19*	*−21*	*−60*	*−9*	*5.12*
**(C) main effect of cue type**							
Middle temporal cortex	R	706	37	48	−63	9	14.62
Middle temporal cortex	L	723	37	−51	−63	6	14.18
Calcarine	L	380	17	−3	−81	−6	12.77
*Calcarine*	*R*		*17*	*6*	*−84*	*−6*	*12.32*
Inferior parietal cortex	R	210	40	36	−42	51	10.54
Inferior parietal cortex	L	316	40	−39	−42	48	7.55
**(D) Interaction among subjects group, cue category and cue type**						
Insula	L	604	47	−30	36	6	4.25
*Caudate*	*L*	*25*	*−21*	*36*	*6*	*4.19*	
*Caudate*	*R*	*47*	*12*	*33*	*−3*	*3.91*	
*Putamen*	*R*	*48*	*18*	*15*	*9*	*3.46*	

*The coordinates (x, y, z) correspond to MNI coordinates. Displayed are the coordinates of the maximally activated voxel within a significant cluster as well as the coordinates of relevant local maxima within the cluster (in italics)*.

**Figure 3:**
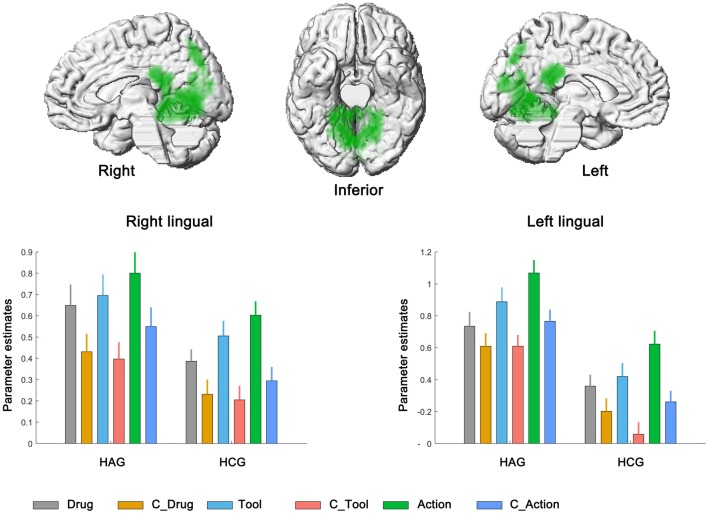
Neural correlates underlying the main effect of cue category. HAG, Heroin abstinent group; HCG, Health control group; C_Drug, neutral drug pictures; C_Tool, neutral tool pictures; C_Action, neutral action pictures.

We then calculated the brain regions activated by the main effect of the cue category. Bilateral lingual showed significantly higher neural activity in drug-related condition than in neutral condition. No significant activation was found in the reverse contrast; see Figure 3, Table 3B.

**Figure 4:**
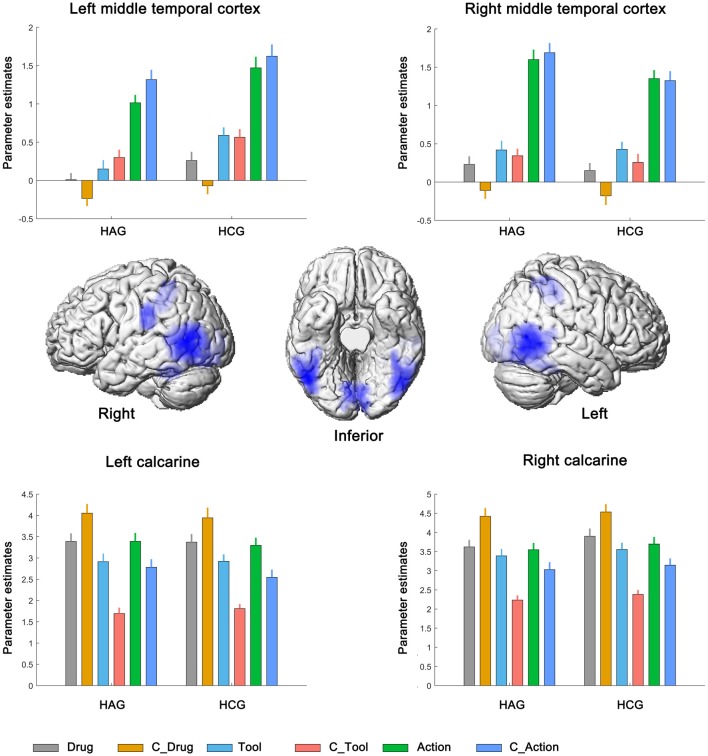
Neural correlates underlying the main effect of cue type. HAG, Heroin abstinent group; HCG, Health control group; C_Drug, neutral drug pictures; C_Tool, neutral tool pictures; C_Action, neutral action pictures.

Finally, bilateral middle temporal cortex (MTC), bilateral calcarine and bilateral IPC showed significantly neural activity associated with the main effect of cue type; see Figure 4, Table 3C. Parameter estimates under the six experimental conditions were extracted from the activated clusters and submitted to a *post hoc* *t*-tests (with Bonferroni’s correction), the results showed that: (1) in bilateral MTC and bilateral IPC, neural activity in condition of Action and C_Action was significantly higher than neural activity in condition of Tool and C_Tool (all *p* < 0.001), neural activity in condition of Tool and C_Tool was significantly higher than neural activity in condition of Drug and C_Drug (all *p* < 0.001); and (2) in bilateral calcarine, neural activity in condition of Drug and C_Drug was significantly higher than neural activity in condition of Action and C_Action (all *p* < 0.001), neural activity in condition of Action and C_Action was significantly higher than neural activity in condition of Tool and C_Tool (all *p* < 0.001).

#### Neural Interaction Among Subjects Group, Cue Category and Cue Type

**Figure 5:**
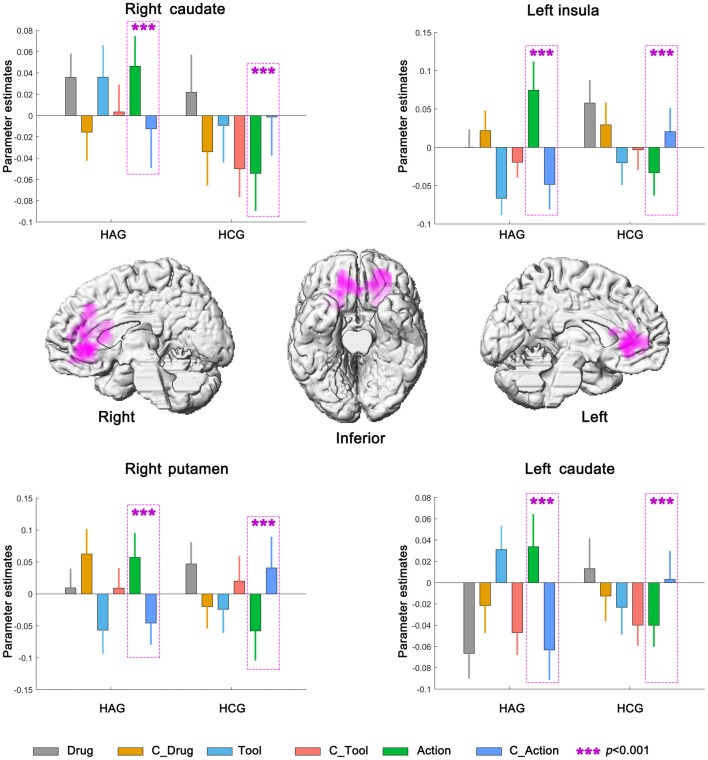
Neural interaction among subjects group, cue category and cue type. HAG, Heroin abstinent group; HCG, Health control group; C_Drug, neutral drug pictures; C_Tool, neutral tool pictures; C_Action, neutral action pictures.

Left insula, bilateral caudate and right putamen were significantly activated in neural interaction among subjects group, cue category and cue type. Parameter estimates under the six experimental conditions were extracted from the activated clusters and submitted to a 2 (subjects group) × 2 (cue category) × 3 (cue type) repeated-measures ANOVA (Planned *t*-tests on simple effects were Bonferroni corrected). For the HAG, Left insula, bilateral caudate and right putamen showed significantly higher neural activity in Action condition than in C_Action condition (all *p* < 0.001); while for the HCG, Left insula, bilateral caudate and right putamen showed significantly lower neural activity in Action condition than in C_Action condition (all *p* < 0.001); see Figure 5, Table 3D.

## Discussion

This study is the first to divide drug-related cues into drug, drug use action and drug use tool, and to use these cues as drug stimuli to induce brain activity. We confirmed that there was activity in different brain regions in response to different drug stimuli, and drug use action cues activated more areas, including the motor area and caudate nucleus (DLS), than the other two types of cues. These results are meaningful because they could explain the mechanism of automatic drug using behavior induced by drug-related cues.

We characterized differences between participants with HAG and HCG in terms of their response to neutral stimuli and to different types of drug-related cues. IFC and IPC, PCC and ACC were observed to be significantly activated for the main effect of subject group, bilateral lingual gyrus was observed to be significantly activated for the main effect of cue category. For HAG, relative to the drug tool and drug cues, the drug use action cues stimulated greater activations in the sensorimotor areas including bilateral MTC, inferior parietal gyrus and calcarine. The neural interaction between participant group, cue category and cue type indicated that the left insula, right caudate and right putamen showed significantly enhanced neural activity in the “HAGaction” condition. These findings partly verified our hypothesis: drug related cues activated both ACC, insula and DSL-sensorimotor circles (IPC, caudate and putamen). The cues related to drug use action activated more of the sensorimotor and dorsal striatum regions.

These results are also in line with some of the addiction literatures, which have repeatedly demonstrated that amygdala, hippocampus, dACC, PFC, components of the mesocorticolimbic system, are innervated by dopaminergic projections from VTA. Activity in these brain regions reflects the reward value predicted by drug related cues (Schultz, 2007; Jasinska et al., 2014). The insula was also activated under drug-related cues, which is often engaged during tasks requiring cognitive control (Lewis, 2006; Stippekohl et al., 2010; Wagner et al., 2011; Jasinska et al., 2014), it contributes to hand-and-eye motor movement and motor learning too (Mutschler et al., 2007). The putamen and caudate nucleus together form the dorsal striatum.The putamen has long been known to be associated with motor processes (Malenka et al., 2009). It is interconnected with many other structures, and works in conjunction with them to influence many types of motor behaviors. A primary function of the putamen is to regulate motor planning and execution (Yager et al., 2015). It was also found to play a role in the “automatic” performance of previously learned movements (Griffiths et al., 1994). This encodes associations between drug-related cues and behavioral responses. It may allow the presentation of drug-related cues to activate automatic behavioral response, which results in drug taking (Goodman and Packard, 2016).

It is known that superior and inferior parietal cortices, posterior MTG and inferior temporal cortex, which are called action-representation regions, store and process action knowledge and tool use skills (Lewis, 2006; Buxbaum et al., 2007) involved in motor preparation and output (Yalachkov and Naumer, 2011). IPL and SPL are also associated with links between and synthesizing of sensory and motor signals (Jacob and Jeannerod, 2005), characterized as a motor resonance system (Rizzolatti and Craighero, 2004). Our study results proved that related cues not only activate the reward-motivation system of addiction which include thalamus, anterior cingulate and insula, but also stimulate activities in the sensorimotor-DLS circuit, which is the neural basis of automatic action response, including inferior and superior parietal gyrus, caudate and putamen.

The Sensorimotor-DLS areas that were activated under different drug-related cues have also been shown to be activated in many other cue-induction studies (Jacob and Jeannerod, 2005; Smolka et al., 2006; Chase et al., 2011; Kühn and Gallinat, 2011; Engelmann et al., 2012; Tang et al., 2012). A meta-analysis of 11 studies pointed out that the more reliably activated regions, including visual system, precuneus, anterior and posterior cingulate cortex, the dorsolateral PFC, insula and dorsal striatum, were activated in most of the fMRI studies on smoke-related cue-induced reaction (Engelmann et al., 2012).

However, little is known about factors that modulate the degree of cue reactivity, especially factors related to the cue itself. It is hard to tell from previous research which specific aspect of the cue induces the specific brain area reactivity, and which area’s activity is the underpinning mechanism of a specific cue. Based on previous research, we distinguished the effect of different cues on cue–induced reactivity. Compared to the other drug cues, the drug use action cues more strongly activated the DLS- Sensorimotor related brain regions. This indicated that drug cues might not be the only essential factor in cue-induced reactivity, especially for behavior reactivity. White (1996) suggested that drugs play the part of “reinforcers” that strengthen associations among drug-related cues and drug using behavior, implying that action representation is possibly the other important mechanism of cue-induced reactivity. Jasinska et al. (2014) argued that drug use history might facilitate the sensorimotor processing of drug cues and promote a suite of learning and plasticity processes. Thus, once addicted individuals are exposed to drug-related cues, automatic drug seeking or using behavior would be elicited. This is coincident with what Tiffany pointed out in 1990: Automatic drug using behavior is assumed to be an important aspect of craving and the key point of relapse.

The current study not only confirmed that the sensorimotor areas associated with action representation are activated under drug-related cues, but moving forward, the study clarifies different types of cues and the effects of each type of cue-induced reactivity. Activations of sensory and motor brain regions in response to drug-associated cues can predict relapse and correlate with craving, severity of dependance and automatized behavioral reactions towards drug-related stimuli (Yalachkov et al., 2009). This highlights the potential use of the cortex representation effect of the sensorimotor system in cue exposure therapy for drug addiction (Choi et al., 2011).

## Conclusion

The findings largely replicate prior neuroimaging research on drug cue reactivity, and also clarify different types of cues’ effects in drug-induced reactivity (Lewis, 2006; Stippekohl et al., 2010; Wagner et al., 2011; Jasinska et al., 2014). The study indicated that drug use action related cues activate corresponding action representations and dorsal striatum in the heroin addicted brain. This finding might be one key to understanding habitual drug using behavior and relapse. The results have important implications not only for a theory of addiction but also for the practical application of neuroscientific findings in the prevention of uncontrolled drug use and the treatment of drug addiction.

## Limitations

A limitation of the study is that static pictures were used as the cues in the research. Even though static cues have proven instrumental to the study of cue-induced craving, dynamic action stimuli may be better suited for studying motor representation. Further research needs to use dynamic action stimuli to confirm the results of this study and to identify the effect of different cues in the process of automatic drug using behavior. Another limitation is that HAG had a higher level of alcohol use on average than HCG, and all participants were permitted to use cigarettes. These factors might have confounded the results (Wagner et al., 2011; Li et al., 2013). However, we do not know if the levels of drinking or smoking in these individuals could explain the results.

## AUTHOR’S NOTE

This is a English language translation/reprint of “Activations of Sensory-motor Brain Regions in Response to Different Types of Drug-associated Cues” originally published in Acta Psychologica Sinica (Zeng et al., 2015). Hong Zeng et al. prepared this translation with support from the NSFC, Education Department of Guangdong Province and Education Department of Guangzhou city. Permission was granted by Acta Psychologica Sinica.

The authors adopted different data analysis method in the current manuscript, which also now include the control group. Additionally, a wider range of opinions is presented in the current manuscript with deeper knowledge of the function of SM-DLS.

## Author Contributions

HZ, QC and HY were responsible for the study design. DS, HZ and MW contributed to the acquisition of fMRI and demographical data. PW performed the data analysis. HZ, PW drafted the manuscript. QC, HY and SV-K provided critical revision of the manuscript. All authors approved the final version of the manuscript for publication and agree to be accountable for all aspects of the work.

## Conflict of Interest Statement

The authors declare that the research was conducted in the absence of any commercial or financial relationships that could be construed as a potential conflict of interest.

## Footnotes

^1^www.fil.ion.ucl.ac.uk/spm/

^2^dbm.neuro.uni-jena.de/cat/

^3^http://sourceforge.net/projects/marsbar
